# Polypharmacy to Mitigate Acute and Delayed Radiation Syndromes

**DOI:** 10.3389/fphar.2021.634477

**Published:** 2021-05-17

**Authors:** Tracy Gasperetti, Tessa Miller, Feng Gao, Jayashree Narayanan, Elizabeth R. Jacobs, Aniko Szabo, George N. Cox, Christie M. Orschell, Brian L. Fish, Meetha Medhora

**Affiliations:** ^1^ Department of Radiation Oncology, Medical College of Wisconsin, Milwaukee, WI, United States; ^2^ Department of Medicine, Medical College of Wisconsin, Milwaukee, WI, United States; ^3^ Department of Physiology, Medical College of Wisconsin, Milwaukee, WI, United States; ^4^ Cardiovascular Center, Medical College of Wisconsin, Milwaukee, WI, United States; ^5^ Department of Veterans Affairs, Research Service, Zablocki VAMC, Milwaukee, WI, United States; ^6^ Institute for Health and Equity, Division of Biostatistics, Medical College of Wisconsin, Milwaukee, WI, United States; ^7^ Bolder BioTechnology Inc., Boulder, CO, United States; ^8^ Department of Medicine, Indiana University School of Medicine, Indianapolis, IN, United States

**Keywords:** polypharmacy, acute radiation syndrome, delayed effects of acute radiation exposure, mitigation, hematopoietic growth factor, lisinopril, supportive care, radiation pneumonitis

## Abstract

There is a need for countermeasures to mitigate lethal acute radiation syndrome (ARS) and delayed effects of acute radiation exposure (DEARE). In WAG/RijCmcr rats, ARS occurs by 30-days following total body irradiation (TBI), and manifests as potentially lethal gastrointestinal (GI) and hematopoietic (H-ARS) toxicities after >12.5 and >7 Gy, respectively. DEARE, which includes potentially lethal lung and kidney injuries, is observed after partial body irradiation >12.5 Gy, with one hind limb shielded (leg-out PBI). The goal of this study is to enhance survival from ARS and DEARE by polypharmacy, since no monotherapy has demonstrated efficacy to mitigate both sets of injuries. For mitigation of ARS following 7.5 Gy TBI, a combination of three hematopoietic growth factors (polyethylene glycol (PEG) human granulocyte colony-stimulating factor (hG-CSF), PEG murine granulocyte-macrophage-CSF (mGM-CSF), and PEG human Interleukin (hIL)-11), which have shown survival efficacy in murine models of H-ARS were tested. This triple combination (TC) enhanced survival by 30-days from ∼25% to >60%. The TC was then combined with proven medical countermeasures for GI-ARS and DEARE, namely enrofloxacin, saline and the angiotensin converting enzyme inhibitor, lisinopril. This combination of ARS and DEARE mitigators improved survival from GI-ARS, H-ARS, and DEARE after 7.5 Gy TBI or 13 Gy PBI. Circulating blood cell recovery as well as lung and kidney function were also improved by TC + lisinopril. Taken together these results demonstrate an efficacious polypharmacy to mitigate radiation-induced ARS and DEARE in rats.

## Introduction

Heightened global tensions have resulted in a worldwide threat of accidental or belligerent radiation exposure. The United States has initiated extensive research for adequate preparedness in case of such events. The National Institute of Allergy and Infectious Diseases (NIAID) developed a program to study the mechanisms of radiation-induced injuries as well as specific countermeasures to mitigate these injuries (DiCarlo et al., 2008; DiCarlo et al., 2011). Ionizing radiation alone can result in a broad spectrum of biological tissue damage and lethality in mammals. Sequential injuries to multiple organs occur after exposure of whole animals to radiation, which patterns radiation-damage observed in humans (Fish et al., 2020). The acute radiation syndrome (ARS) occurs first, with gastrointestinal (GI) injury starting within a week after exposure followed by bone marrow toxicity in rodents, nonhuman primates (NHP) and humans (Fliedner et al., 2005; Unthank et al., 2015; Fish et al., 2020). Survivors of ARS proceed to develop the delayed effects of acute radiation exposure (DEARE) which manifest as multiple, spatial sequelae including lung injury (radiation pneumonitis after ∼42-days) and kidney injury (radiation nephropathy after ∼120-days), depending on the initial radiation dose. In rodents, other organs such as the brain, heart, etc. also manifest DEARE but these injuries are lethal at PBI doses higher than those that cause lethality by bone marrow or lung toxicities (Moulder, 2014; Boerma et al., 2016). Approval of drugs to mitigate such radiation injuries requires pivotal efficacy screening through the Food and Drug Administration (FDA) Animal Rule, using animal models that manifest responses similar to humans (USFDA, 2015b). Therefore, in order to identify mitigators for radiation injuries we have developed rat models to simulate the damage from exposure to near total body volumes. Following single high exposures to radiation to the total body in WAG/RijCmcr rats (equivalent to 5–12 Gy in humans), ARS occurs within the first 30-days. This syndrome covers gastrointestinal injury (days 3–7) and hematopoietic cell depletion (days 8–30). Partial bone marrow shielding (5–8%), and supportive care are needed for rats to survive ARS past 30-days, at doses >12.5 Gy, after which they will experience DEARE, with damage to the lungs, kidneys, and other organs. Lung injury can be fatal at 13 Gy or higher and occurs between days 40–90 while lethal renal injury manifests after doses as low as 8–9 Gy (Moulder et al., 2011), but after more than 120-days (Fish et al., 2016).

Currently, several agents have demonstrated efficacy to mitigate ARS (Ng et al., 2020; REMM, 2020). However, only granulocyte colony-stimulating factor (G-CSF, Neupogen), granulocyte macrophage colony-stimulating factor (GM-CSF, Leukine) and PEGylated G-CSF (Neulasta) are approved by the U.S. Food and Drug Administration (FDA) to be used after exposure to myelosuppressive doses of radiation (USFDA, 2015a; USFDA, 2021; Amgen, 2015a; Amgen, 2015b; Sanofi-Aventis, 2018). Recently, Nplate (romiplostim) has also been approved by the FDA to be used after similar doses of radiation for the treatment of thrombocytopenia (Amgen, 2021; USFDA, 2021). These cytokines are kept in the Strategic National Stockpile which is managed by the U.S. Department of Health and Human Services and the Office of the Assistant Secretary for Preparedness and Response (REMM, 2020).

Another promising hematopoietic growth factor (HGF) is Interleukin (IL)-11, a member of the IL-6-type cytokine family (Lee et al., 2012). It is approved to treat chemotherapy-induced thrombocytopenia. IL-11 also protects against renal injury in mice, human proximal tube injury in culture, and attenuates the inflammatory responses in a murine model of lipopolysaccharide-induced sepsis.

In addition, PEGylated GM-CSF and PEG-IL11 have been shown in rodents to possess longer half-lives and induce longer-lasting increases in hematopoietic cells through neutrophil recovery and their ability to increase immune function in rodents (Plett et al., 2014; Kumar et al., 2018; Cox et al., 2020). We used a combination of these PEG-HGF mitigators (PEG-GM-CSF, PEG-G-CSF, and PEG-IL-11, Bolder Biotechnology Inc., 2425 55th St., Suite 210, Boulder, CO 80301 United States) for the current study, and will refer to them as a triple combination (TC). The TC included a PEGylated murine (m) derivative of GM-CSF (PEG mGM-CSF), to closely match species specificity observed for GM-proteins. However, PEGylated human (h) derivatives of G-CSF (PEG hG-CSF) and IL-11 (PEG hIL-11) were included in the TC, since they are known to be bioactive in rodents.

ACE inhibitors are one of few mitigators for DEARE in rats. The use of an ACE inhibitor significantly decreased morbidity caused by pneumonitis (Kma et al., 2012) even if started 35-days after irradiation (Gao et al., 2013). The ACE inhibitor lisinopril decreased renal injury in rats given 13 Gy leg-out partial body irradiation (leg-out PBI) (Fish et al., 2016). Leg-out PBI exposes the whole body to irradiation, except for part of one hind leg that is shielded. This allows for bone marrow repopulation to allow DEARE to manifest in the lung and kidneys without bone marrow transplantation. In addition, ACE inhibitors including lisinopril, reduced the prevalence of radiation-induced pneumonitis in cancer patients treated with radiotherapy (Jenkins and Watts, 2011; Jenkins and Welsh, 2011; Kharofa et al., 2012), indicating efficacy in humans. However, ACE inhibitors have not shown efficacy to mitigate ARS in rats (Fish et al., 2016) but are efficacious in mice (Davis et al., 2010; Barshishat-Kupper et al., 2011; McCart et al., 2019), which necessitates the evaluation of combining ACE inhibitors with other promising mitigators.

Our ultimate goal is to treat both ARS and DEARE. Since no single agent has been identified, we are developing a compatible, multi-agent approach. We are combining promising mitigators to demonstrate efficacy for at least four sequelae that occur after irradiation. Female WAG/RijCmcr rats given leg-out PBI provide some of the best characterized models available for such studies. These models were used in this initial proof of concept study. Future studies with adult, geriatric and pediatric models using male and female rats will help confirm the efficacy of this approach for FDA approval.

## Methods

### Animal Care

All animal use was approved by the Institutional Animal Care and Use Committees (IACUC) at the Medical College of Wisconsin and care for the rats was provided as previously described (Fish et al., 2016). All rats were fed Teklad 8904 diet and provided reverse osmosis (RO) hyper-chlorinated water ad libitum. In order to study the efficacy of a combination of drugs for ARS and DEARE, two sets of experiments using different injury models were used.

### Acute Radiation Syndrome Study

#### Total Body Irradiation in Rats

Female WAG/RijCmcr rats (11–12 weeks of age) were given total body irradiation (TBI) without the use of anesthetics. All rats were placed in a plastic jig and were irradiated using a XRAD 320 KV orthovoltage x-ray system (Precision X-Ray, North Branford, Connecticut) as previously described (Medhora et al., 2019). The X-ray system was operated at 320 kVp and 13 mAs with a half value layer of 1.4 mm Cu and a dose-rate of 1.75 Gy min^−1^ for a total dose of 7.5 Gy.

#### Dosimetry for Irradiation (Medhora et al., 2014)

A Soft X-Ray ionization Chamber (PTW, Germany) was used to collect depth dose information. Absolute calibration measurements were made using a Farmer-type ionization chamber and a Kiethley electrometer. This system was calibrated for the orthovoltage energy range at the Accredited Dosimetry Calibration Laboratory located at University of Wisconsin, Madison, WI. Measurements performed in this laboratory are directly traceable to the National Institute of Standards and Technology. The ionization was measured in air and then converted to absolute dose in water following the American Association of Physicists in Medicine Task Group-61 protocol (Ma et al., 2001). The dose rate for TBI was defined at the midline of the rat and was calculated as described for TBI and leg-out PBI using measured output of the machine and the depth dose data. Then the irradiation time, including appropriate timer error of the X-ray machine, was calculated to deliver the required dose in one fraction using a posterior-to-anterior beam. Gafcromic film EBT2 (ISP, United States) sandwiched between slabs of solid water phantom was used to obtain profile distributions. The dose at the centers of the two rat chambers varied by 2%, and rats were randomly assigned to chambers to avoid any resulting bias. The irradiation field at midline was large enough to cover both chambers with adequate (at least 2 cm) margins.

#### Interventions

**FIGURE 1:**
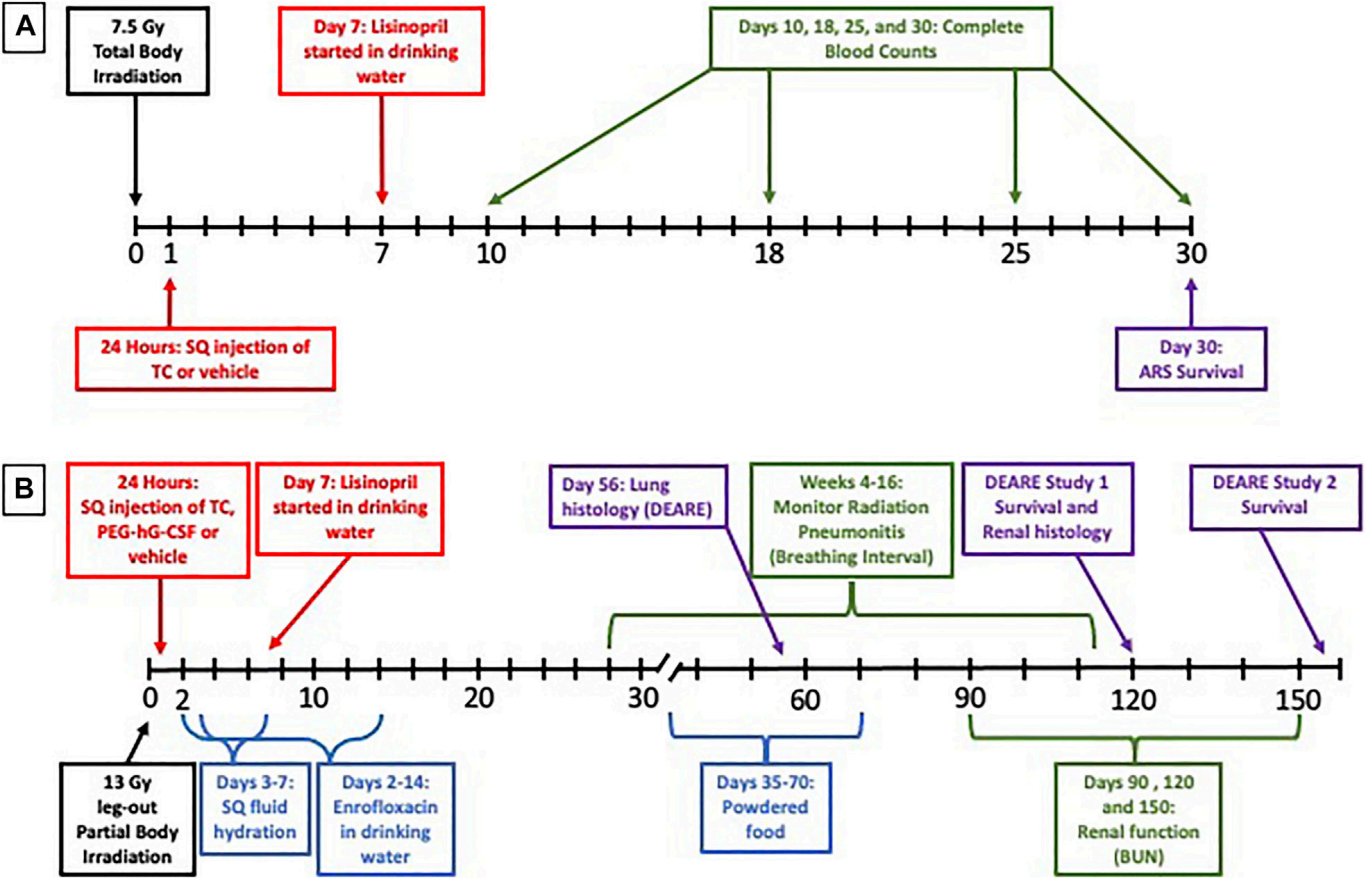
Experimental design. To study the Acute Radiation Syndrome (ARS) **(A)**, female WAG/RijCmcr rats (11–12 weeks of age) were irradiated with 7.5 Gy total body irradiation (TBI). At 24-h post-irradiation, all rats received a subcutaneous (SQ) injection of either the triple combination (TC, consisting of PEG-hG-CSF, PEG mGM-CSF and PEG hIL-11) or vehicle and the ACE inhibitor lisinopril was started in the drinking water (24 mg m^−2^ d^−1^) in two groups at 7 days post-irradiation. A subset of each treatment group was bled at days 10, 18, 25, and 30-days and complete blood counts were analyzed. The ARS experiment was terminated at 30-days.To study the Delayed Effects of Acute Radiation Syndrome (DEARE) **(B)**, female WAG/RijCmcr rats (11–12 weeks of age) were irradiated with 13 Gy leg-out partial body irradiation (leg-out PBI). At 24-h post-irradiation, all rats received a subcutaneous (SQ) injection of either the triple combination (TC, consisting of PEG-hG-CSF, PEG mGM-CSF and PEG hIL-11), PEG-hG-CSF or vehicle and the ACE inhibitor lisinopril was started in the drinking water (24 mg m^−2^ d^−1^) in two groups at 7 days post-irradiation. Supportive care consisting of antibiotics (enrofloxacin, 10 mg kg^−1^ d^−1^, days 2–14) in the drinking water, SQ fluid hydration (saline, 40 ml kg^−1^ d^−1^, days 3–7) and supplemental powdered diet (days 35–70) were provided to all rats in the DEARE study. Radiation pneumonitis was monitored weekly by recording breathing rates (weeks 4–16) and while radiation nephropathy was monitored by measuring the blood urea nitrogen (BUN, days 90, 120, and 150). Lungs and kidneys were harvested at 56 and 120 days respectively to observe lung and renal radiation injury. The DEARE studies were terminated at 120 or 160-days.

The experimental design of the ARS study can be visualized in Figure 1A. After irradiation, rats were randomly assigned to one of five study arms: 1) 7.5 Gy TBI (*n* = 27); 2) 7.5 Gy TBI + vehicle (*n* = 41); 3) 7.5 Gy TBI + TC (*n* = 39); 4) 7.5 Gy TBI + vehicle + lisinopril (*n* = 27); or 5) 7.5 Gy TBI + TC + lisinopril (*n* = 36). A group of age-matched non-irradiated controls (*n* = 12) were also included in this study. A single dose of either TC (2.75 ml kg^−1^, 10 mM Sodium Phosphate, 4% Mannitol, 1% Sucrose; PEG hG-CSF 0.55 mg kg^−1^; PEG mGM-CSF 0.55 mg kg^−1^; PEG hIL-11 0.165 mg kg^−1^) (Bolder BioTechnology, Boulder, CO) or the matched vehicle (2.75 ml kg^−1^, 10 mM Sodium Phosphate, 4% Mannitol, 1% Sucrose) was subcutaneously injected into assigned groups 24-h post-irradiation. At 7-days post-irradiation, when recovery from GI toxicity is usually observed, lisinopril (21CEC PX Pharm Ltd. United Kingdom; 24 mg m^−2^ d^−1^) was started in the drinking water and continued until termination (groups 4 and 5). Secondary endpoints for GI-ARS were not included in the protocol because the dose of radiation in the TBI model (7.5 Gy) was well below the threshold to observe non-invasive symptoms of GI injury such as diarrhea (doses >11 Gy in WAG/RijCmcr rats, Fish et al., 2020). The TC were not expected to alter GI-ARS based on other studies with the components of the TC (Chua et al., 2014; Cox et al., 2020). Whole blood was collected via the jugular vein at days 10-, 18-, 25-, and 30-post-irradiation. The experiment was terminated at 30-days and the rats were euthanized.

#### Blood Cell Counts

A subset of rats from each study arm including: 1) 7.5 Gy TBI control (*n* = 12), 2) 7.5 Gy TBI + vehicle (*n* = 11), 3) 7.5 Gy TBI + TC (*n* = 11), 4) 7.5 Gy TBI + vehicle + lisinopril (*n* = 11), or 5) 7.5 Gy TBI + TC + lisinopril (*n* = 11), were anesthetized with 3–5% isoflurane and bled via jugular vein by a trained technician (Medhora et al., 2019). EDTA was used to prevent blood clotting. Whole blood was sent to Marshfield Laboratories (Marshfield, WI) for complete blood counts (CBC). Hematocrit, neutrophils, platelets, red blood cells, percent reticulocytes, and absolute reticulocytes were analyzed to monitor hematopoietic injury.

#### Statistical Analyses

Analysis for 30-day morbidity was shown by Kaplan-Meier plots and tested for differences between groups by Cox regression. Neutrophil counts were analyzed using linear mixed effects models with a random animal intercept to account for repeated measures. Based on a Box-Cox analysis, the counts were log-transformed for analysis because this step improved the linearity of the effects and the normality and homoskedasticity of the residuals. The results were summarized by pairwise comparison of treatments within each day and were adjusted for multiple testing within each time-point using Tukey’s method. Analyses were performed using R 3.5.0 (R Foundation for Statistical Computing, Vienna, Austria).

### Delayed Effects of Acute Radiation Exposure Study

#### Leg-Out Partial Body Irradiation in Rats

Female WAG/RijCmcr rats (11–12 weeks of age) were given leg-out PBI without the use of anesthetics as previously described (Medhora et al., 2019). This model of irradiation exposes the entire body of the rat to ionizing radiation except for one hind limb which is out of the field. Therefore, it is referred to as leg-out PBI rather than TBI since the entire rat is not exposed to radiation. Briefly, rats were immobilized in a plastic jig and irradiated in the same manner as described for TBI, to a total dose of 13 Gy. To allow for bone marrow recovery, one hind limb of each rat was carefully externalized from the jig and shielded from radiation with a 0.25-inch lead block. The dose to this leg was ∼2 Gy (5–8% bone marrow shielding). Dosimetry was conducted as previously described for the Acute Radiation Syndrome study (Medhora et al., 2014; Medhora et al., 2015).

#### Interventions

The experimental design of the DEARE experiments can be visualized in Figure 1B. All rats were given supportive care of enrofloxacin (10 mg kg^−1^ d^−1^) from days 2–14 post-irradiation and subcutaneous fluid hydration (saline, 40 ml kg^−1^ d^−1^) from days 3 to 7 post-irradiation, as described previously (Fish et al., 2016). To study DEARE to 120-days, female rats were randomly assigned to one of five study arms: 1) Non-irradiated + vehicle (for TC, *n* = 10); 2) 13 Gy leg-out PBI + vehicle (*n* = 28); 3) 13 Gy leg-out PBI + TC (*n* = 21); 4) 13 Gy leg-out PBI + PEG-hG-CSF (*n* = 21); or 5) 13 Gy leg-out PBI + TC + lisinopril (*n* = 16). To study DEARE to 160-days, female rats were randomly assigned to one of the following four study arms: 1) 13 Gy leg-out PBI + vehicle (for TC) (*n* = 12); 2) 13 Gy leg-out PBI + TC (*n* = 12); 3) 13 Gy leg-out PBI + vehicle + lisinopril (*n* = 12); or 4) 13 Gy leg-out PBI + TC + lisinopril (*n* = 12). Since lisinopril is soluble in water, no vehicle specific for lisinopril was required. A single dose of either TC, vehicle for TC (see ARS study) or PEG-hG-CSF (BBT-015 0.55 mg kg^−1^, Bolder BioTechnology, Boulder, CO) was subcutaneously injected into assigned groups 24 h post-irradiation. At 7-days post-irradiation, lisinopril (21CEC PX Pharm Ltd. United Kingdom; 24 mg m^−2^ d^−1^) was started in the drinking water and continued until termination in one group. All rats provided powdered diet in addition to pelleted diet days 35–70 post-irradiation due to tooth loss following leg-out PBI. Tooth loss is observed when the head of rats is not shielded during exposure, but the teeth grow back by day 70. The supplemental powdered food eliminates weight loss due to the inability to eat pelleted food.

Since lethal GI injury occurs at doses >11 Gy leg-out PBI (Fish et al., 2020) without supportive care and above 13 Gy with supportive care, secondary endpoints for GI toxicity were not included. For the 120-days DEARE study, breathing rates (BR) were recorded every other week starting at week 4 post-irradiation and continuing through week 16 to evaluate lung function. Blood was collected via jugular vein at 90- and 120-days post-irradiation to monitor blood urea nitrogen (BUN) levels. BUN is a measure of renal function. For the 120-days study, at day 56 (during radiation pneumonitis), random sets of rats from each study arm were euthanized for lung histology (peak of pneumonitis). At termination (day 120), rats were euthanized, and the kidneys harvested for histology.

The second DEARE study was terminated at 160-days, with survival serving as the primary end point. BUNs were measured at 90-, 120- and 150-days post-irradiation.

Blood was harvested, followed by necropsy for all rats that were identified as moribund to confirm morbidity due to lethal radiation pneumonitis or nephropathy as the cause of death.

#### Measurement of Breathing Interval

To monitor radiation pneumonitis, breathing rates and body weights were measured every other week from weeks 4 to 16, as previously described (Medhora et al., 2014; Medhora et al., 2015). Rats were placed in a plastic restrainer for 5 min for two consecutive training days to allow the rats to become acclimated to the apparatus. On the third day, the restrainer was placed in a transparent EMKA plethysmograph (Scireq Scientific Respiratory Equipment Inc., Montreal, QC, Canada) which measured the frequency of pressure changes. Each rat was recorded for a maximum of 10 min and the mean breathing rate was calculated from four steady 15 s recordings. The inverse of the breathing rates was calculated to derive the breathing interval or time/breath in minutes. Higher breathing rates and lower breathing intervals are associated with more lung damage. The breathing interval was set to 0 for all animals that were moribund during pneumonitis to account for attrition (Medhora et al., 2012; Gao et al., 2014; Medhora et al., 2020).

#### Measurement of Blood Urea Nitrogen

A sensitive method to assess radiation-induced nephropathy is to measure the serum BUN levels which correlate well with renal histopathology as previously published (Moulder et al., 2011). Rats were anesthetized with isoflurane (3–5%) and blood was drawn via the jugular vein by a trained technician at days 90 and 120 post-irradiation (Medhora et al., 2019). The BUN was assayed from serum as described previously (Cohen et al., 1994; Medhora et al., 2014; Fish et al., 2016) using a urease-nitroprusside colorimetric assay. BUN values were expressed as mg dL^−1^ of serum and medians with 20–80% ranges were used for statistical analysis. Irradiated rats with BUN > 120 mg dl^−1^ had lethal radiation nephropathy and were euthanized and given a value of 120 mg dl^−1^ to account for attrition, since such rats were previously confirmed to have severe and irreversible renal damage (Moulder et al., 1993; Fish et al., 2016; Medhora et al., 2020).

#### Lung Histology

A subset of irradiated rats from the 120-days DEARE study was assigned for lung histology at 56-days after 13 Gy leg-out PBI as described previously (Medhora et al., 2014; Medhora et al., 2015). Briefly, the lungs were harvested, inflated, and fixed by gravity using 10% buffered formalin (Fisher Scientific, Pittsburg, PA) and the left lung was embedded in paraffin. Whole mount left lung sections (4 µm thick) were stained with H&E. Five (20×) fields from each rat were randomly selected and scored by operators blinded to the treatment groups. Vessel wall thickness, alveolar wall thickness, and foamy macrophages were scored as described previously (Medhora et al., 2014; Medhora et al., 2015). Higher scores indicated more severe lung injury.

#### Kidney Histology

At the termination of the study (120-days post 13 Gy leg-out PBI), the kidneys were harvested, cut into halves and immediately fixed in 10% buffered formalin and processed for paraffin embedding. Kidney sections were stained with H&E, and the kidney injury blinded and assessed in coded samples as described earlier (Moulder et al., 1993). Kidneys were scored as follows: absence of renal cyst (0); presence of microscopic (1+); and macroscopic (2+) cysts. Glomerular sclerosis was assessed by studying 20 random glomeruli per slide as follows: 1–2 sclerosed glomeruli (1+); 3–4 sclerosed glomeruli (2+); or 5 or more sclerosed glomeruli (3+). Interstitial fibrosis was assessed on an increasing scale as none (0); scattered (1+); or diffuse (2+). Glomerular mesangiolysis was assessed as absent (0); variably present (1+); present in most glomeruli (2+); and present in all glomeruli (3+). These scores were then aggregated to get a composite histologic score. Higher scores indicated more severe renal injury.

#### Statistical Analyses

Analysis for morbidity after 30-days is shown by Kaplan-Meier plots and the three growth-factor treated groups were analyzed using Cox regression, with pairwise comparisons using a multivariate normal distribution-based single-step adjustment for multiple comparison control. Breathing intervals are shown as means with 95% CIs. BUN values are shown as medians and 20–80% ranges. Statistical differences of breathing intervals and BUN values were calculated by the ANOVA on Ranks with multiple comparisons by the Dunnett’s method and both accounted for attrition. For analyses of histological results, a one-way ANOVA was used to determine significance. All pairwise multiple comparisons were conducted with the Holm-Sidak method as post-hoc analysis. In case data failed either normality or equal variance tests, ANOVA on ranks with all pairwise multiple comparisons by Dunn’s method were used.

## Results

### Mitigation of Acute Radiation Syndrome

#### Enhanced Survival After 7.5 Gy TBI With PEG-HGFs

**FIGURE 2:**
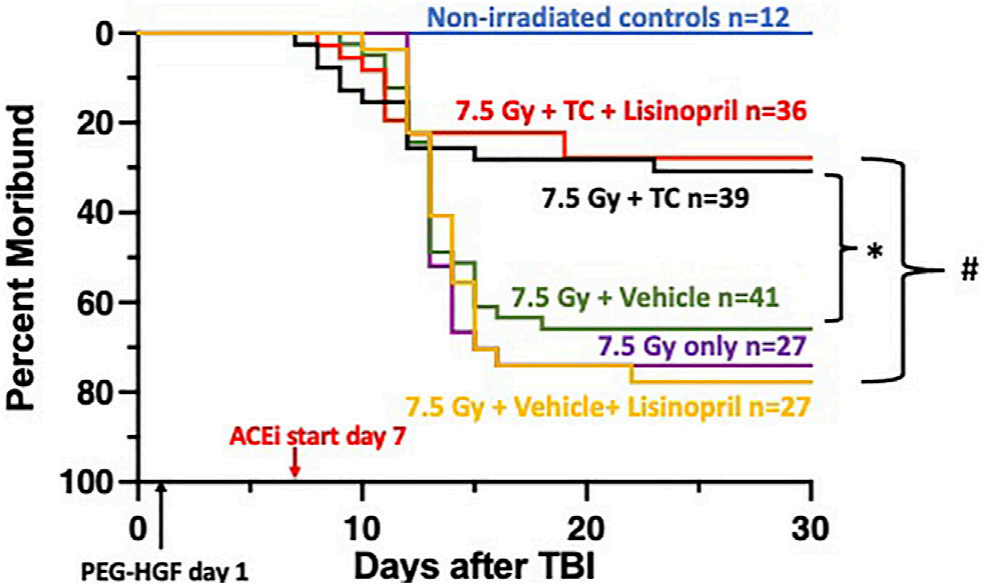
Mitigation of hematopoietic-acute radiation syndrome (H-ARS) by triple combination with and without lisinopril. Kaplan-Meier plots show morbidity through 30-days after 7.5 Gy total body irradiation (TBI). The triple combination (TC, consisting of PEG-hG-CSF, PEG mGM-CSF and PEG hIL-11) or vehicle were given subcutaneously 24-h after TBI (designated by PEG-HGF) and the ACE inhibitor, lisinopril, was started in the drinking water 7 days after irradiation. The number of rats in each group is designated by the “*n*.” Non-irradiated controls are represented with the blue line. Morbidity was not different in the three irradiated groups given 7.5 Gy only, with vehicle or lisinopril, but survival was enhanced in the group which received the TC (*p* = 0.05, denoted by * compared to 7.5 Gy + vehicle group). Survival was increased in the irradiated group receiving TC and lisinopril compared to the irradiated rats receiving the vehicle for TC and lisinopril (*p* < 0.05, denoted by #).

Rats were irradiated with 7.5 Gy TBI at 11–12 weeks of age and randomly assigned to one of five treatment groups to assess morbidity due to hematopoietic injury (see *Methods*). At 24-h post-irradiation, rats were injected subcutaneously with TC or vehicle. The ACE inhibitor, lisinopril, was started in the drinking water 7-days post-irradiation in two irradiated groups, one that received TC and one that received the vehicle for TC. Figure 2 shows a Kaplan-Meier survival plot to 30-days post-irradiation, through hematopoietic acute radiation syndrome (H-ARS) for the five treatment groups and age-matched, non-irradiated controls. At 30-days post-irradiation, 69% of the rats that received 7.5 Gy TBI, but no TC, were moribund. The addition of the TC improved survival (*p* = 0.05) compared to irradiated animals receiving the vehicle only. The rats that received 7.5 Gy TBI with TC and lisinopril also had enhanced survival as compared to the vehicle and lisinopril group, with only 28% morbidity (*p* < 0.05). Therefore, TC improves survival, even in the presence of lisinopril. None of the non-irradiated control rats were moribund in this study.

#### Effects of PEG-HGFs and Lisinopril on Recovery of blood BCell Counts After Radiation

**FIGURE 3:**
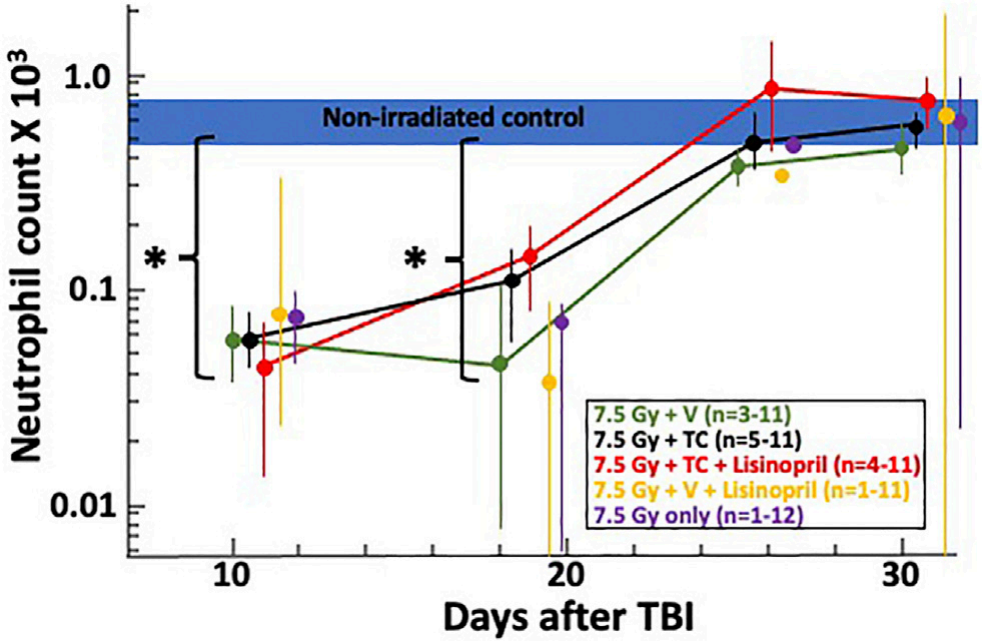
Neutrophil recovery through 30-days post 7.5 Gy total body irradiation (TBI) is shown on a log-scale for the neutrophil count (×10^3^). The horizontal blue bar represents the neutrophil counts for non-irradiated controls. Asterisks (*) represent *p* < 0.02 as compared to non-irradiated controls. At 18 days, the two irradiated groups given the triple combination (TC, consisting of PEG-hG-CSF, PEG mGM-CSF and PEG hIL-11) with (red) and without lisinopril (black), had higher neutrophil counts (*p* ≤ 0.005) than the irradiated groups given the vehicle and lisinopril (yellow). Neutrophil counts returned to levels resembling non-irradiated controls by 25-days post-irradiation. The “*n*” values represent the number of rats alive in each group between 10 and 30 days after irradiation which decreased over time due to rats becoming moribund. Error bars represent 95% CIs for the mean.

Complete blood cell counts at 10-, 18-, 25- and 30-days post-irradiation were measured as a secondary endpoint to examine the bone marrow injury after TBI. Blood collection began at 10-days post-irradiation since this is typically when hematopoietic injury is observed in this model. Figure 3 shows at 10-days the neutrophil count had dropped for all irradiated groups compared to the control (non-irradiated) group (denoted by a blue bar). At day 18, neutrophils in all irradiated groups were still lower than in the control group. Neutrophils in the irradiated groups given the TC, with or without lisinopril, were significantly higher than irradiated rats given the vehicle and lisinopril (*p* ≤ 0.005). All irradiated groups were not different from control values by day 25. Platelet counts were not reported due to inconsistent reporting in a number of samples.

### Mitigation of Delayed Effects of Acute Radiation Exposure to 120 days

#### Survival After 13 Gy Leg-Out PBI With Lisinopril

**FIGURE 4:**
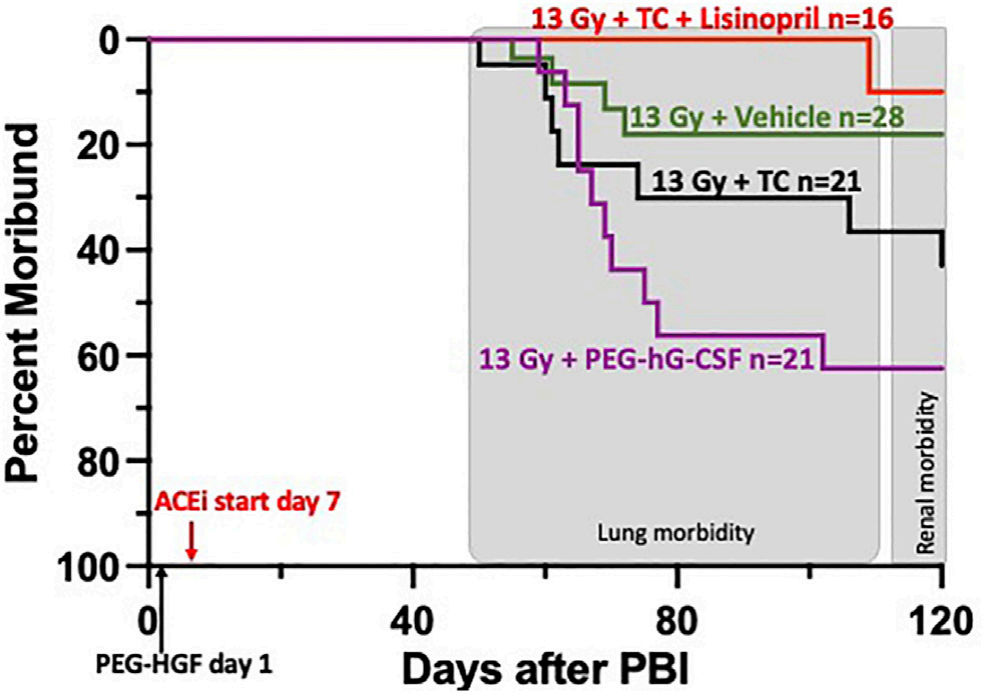
Kaplan-Meier plot representing morbidity from DEARE up to 120 days post 13 Gy partial body irradiation with one hind limb shielded (leg-out PBI). The triple combination (TC, consisting of PEG-hG-CSF, PEG mGM-CSF and PEG hIL-11), vehicle or PEG-hG-CSF (BBT-015) were given subcutaneously 24 h post leg-out PBI (designated by PEG-HGF) and the ACE inhibitor, lisinopril, was started in the drinking water 7 days post irradiation (24 mg m^−2^ d^−1^). All irradiated rats were given supportive care with subcutaneous hydration (40 ml kg^−1^ d^−1^) and enrofloxacin (10 mg kg^−1^ d^−1^) from days 3–7 to 2–14, respectively,. There was trend in lower morbidity in irradiated rats that received TC + lisinopril compared to irradiated rats that received PEG-hG-CSF, but this was not significant (*p* = 0.07). Shaded (gray) areas represent the timing for the lung and renal injuries.

Rats were irradiated with 13 Gy leg-out PBI at 11–12 weeks of age and randomly assigned to one of four treatment groups (see *Methods* and Figure 4) to assess survival through DEARE to 120-days. Since PEG-G-CSF (Neulasta, Amgen) is an approved medical countermeasure for ARS, we tested Bolder BioTechnology’s PEG-hG-CSF (BBT-015, one of the components of the TC) alone to determine if it altered morbidity during DEARE. The study was terminated at 120-days and Figure 4 shows a Kaplan-Meier survival plot for these four treatment groups. By 120-days, 63% of the irradiated rats given PEG-hG-CSF only were moribund, with the majority occurring between 60 and 80 days coinciding with pneumonitis (Fish et al., 2016). There was morbidity in all groups starting at 50-days post-irradiation except in the irradiated rats that received TC + lisinopril (6% morbidity). Though the addition of lisinopril trended to increase survival to 120-days in the irradiation rats given TC, this did not reach significance from the 13 Gy + PEG-hG-CSF only group (*p* = 0.07).

#### Mitigation of Radiation Pneumonitis With Lisinopril

##### Breathing Interval Measurements to Monitor Lung Injury During Pneumonitis

**FIGURE 5:**
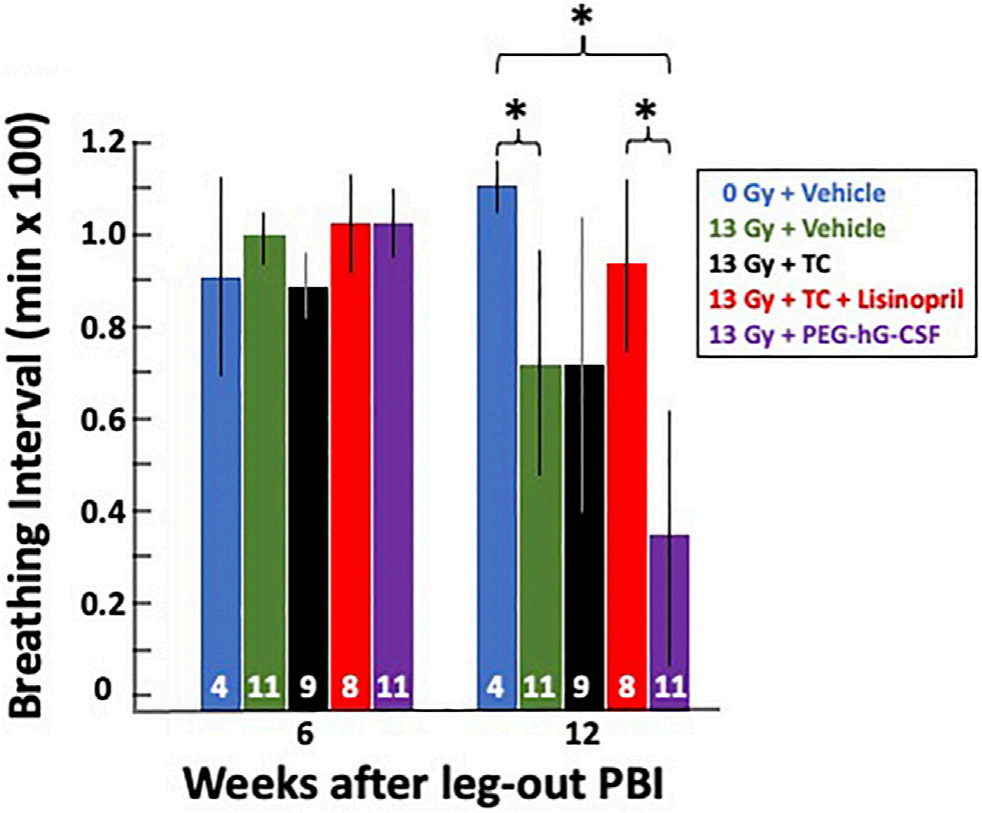
Radiation pneumonitis is mitigated by lisinopril after 13 Gy leg-out PBI. Graphical representation of breathing intervals as a secondary and functional endpoint for lung injury were obtained from all 13 Gy leg-out PBI irradiated treatment groups and non-irradiated controls. Breathing rates were recorded biweekly from weeks 4 to 16 post-irradiation and converted to breathing intervals (see *Methods*). The bars represent means with 95% CIs. Rats moribund with lung injury confirmed at necropsy (13 Gy + vehicle *n* = 2, 13 Gy + PEG-hG-CSF *n* = 6, 13 Gy + TC *n* = 2) were given a breathing interval of 0 at 12 weeks to account for attrition. Numbers in the bars represent N in each group. Asterisks represent *p* < 0.05 between treatment groups at 12 weeks, in brackets: 0 Gy + vehicle vs. 13 Gy + PEG-hG-CSF, 0 Gy vehicle vs. 13 Gy + vehicle, 13 Gy + TC + lisinopril vs. 13 Gy + PEG-hG-CSF.

As a secondary endpoint and to monitor the progression of pneumonitis, breathing rates were recorded biweekly starting at week 4 until week 16 (see *Methods*). The breathing rates were then converted to breathing intervals (1/breathing rates in min/breath) to account for attrition from lethal pneumonitis (see *Methods*). Figure 5 shows the mean breathing intervals of each group at 6-and 12-weeks post-irradiation with 95% CIs. There was no difference in breathing intervals at the 6-weeks time point prior to the onset of pneumonitis. All irradiated groups had lower breathing intervals compared to non-irradiated rats at 12-weeks which correlates to the peak of radiation induced lung injury (radiation pneumonitis). The treatment group receiving the vehicle and also the group given PEG-hG-CSF had significantly decreased (*p* < 0.05) breathing intervals when compared to the non-irradiated controls at 12-weeks. This was not observed in the irradiated group given the TC. The irradiated group given PEG-hG-CSF also had significantly decreased (*p* < 0.05) breathing intervals when compared to the treatment group given TC and lisinopril. The addition of lisinopril to TC but not TC alone, mitigated the radiation-induced lung injury that was observed with PEG-hG-CSF alone at 12-weeks.

##### Histological Changes at 56-days to Determine Lung Injury During Pneumonitis

**FIGURE 6:**
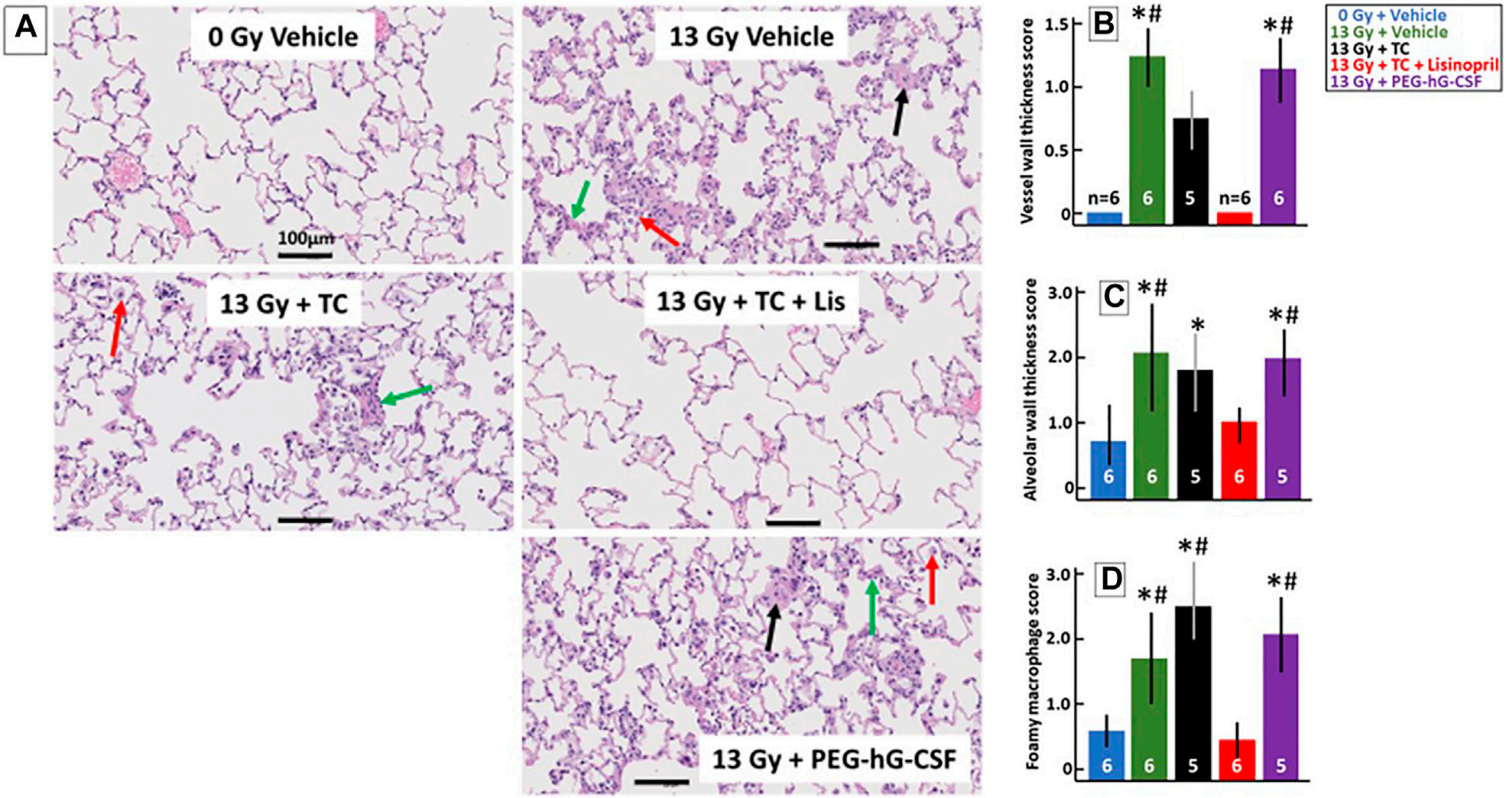
Lisinopril mitigates histological lung injury after 13 Gy leg-out PBI. Representative histological sections of lung tissue harvested at 56-days after 13 Gy leg-out PBI were stained with H&E for each treatment group **(A)**. Irradiated rats given the vehicle or PEG-hG-CSF showed increased alveolar wall thickness cellularity (green arrow), increased vessel wall thickness (black arrow) and foamy macrophages (red arrow). Black bars represent 100 μm. Graphical representations of the H&E-stained lung sections are shown for vessel wall thickness **(B)**, alveolar wall thickness **(C)** and foamy macrophages **(D)**. Vessel wall thickness **(B)** were increased in irradiated rats given the vehicle or PEG-hG-CSF, whereas TC + lisinopril mitigated lung injury (*p* < 0.05, denoted by * compared to controls and # compared to TC + lisinopril). Irradiation increased alveolar wall thickness **(C)** in all irradiated groups compared to control (*p* < 0.05, denoted by *) except for the TC + lisinopril group. Alveolar wall thickness was also increased compared to the TC + lisinopril group in the irradiated vehicle and PEG-hG-CSF groups (*p* < 0.05, denoted by #). Foamy macrophages **(D)** were significantly increased in irradiated rats given the vehicle, TC or PEG-hG-CSF (*p* < 0.05, denoted by * compared to controls and # compared to TC + lisinopril). Numbers in the bars represent N in each group and bars are means with standard deviations.

Histological changes in the lungs during pneumonitis were monitored in a subset of rats at 56-days post-irradiation after lung inflation and staining with H&E (see *Methods*). Figure 6A shows representative lungs from each group that were scored on three characteristic histological changes in irradiated lungs: vessel wall thickness (black arrow), alveolar wall thickness (green arrow) and foamy macrophages (red arrow). Non-irradiated lungs had a lacy architecture with open alveolar spaces, thin walls, patent blood vessels and few macrophages. The irradiated lungs had more congestion and many more infiltrating cells with tissue damage as compared to the non-irradiated lungs, except for the rats that received the TC + lisinopril (red bars, Figure 6B). These lungs were more comparable to control lungs with morphometric measurements indicating lower histological scores (Figures 6B–D). Vessel walls (Figure 6B) were significantly thicker in 13 Gy vehicle and 13 Gy PEG-hG-CSF groups compared to non-irradiated lungs and 13 Gy TC + lisinopril groups (*p* < 0.05). Alveolar wall thickness (Figure 6C) increased in all irradiated groups, but was significantly different in the vehicle, TC and PEG-hG-CSF groups but not the TC + lisinopril groups compared to the lungs from non-irradiated rats. Foamy macrophages (Figure 6D) were abundant in all irradiated groups, except for the TC + lisinopril group, which was not different from the non-irradiated group lungs. These results show histological injuries in irradiated lungs which were mitigated with TC + lisinopril.

#### Mitigation of Radiation Nephropathy With Lisinopril

##### Blood Urea Nitrogen Measurements to Monitor Renal Injury

**FIGURE 7:**
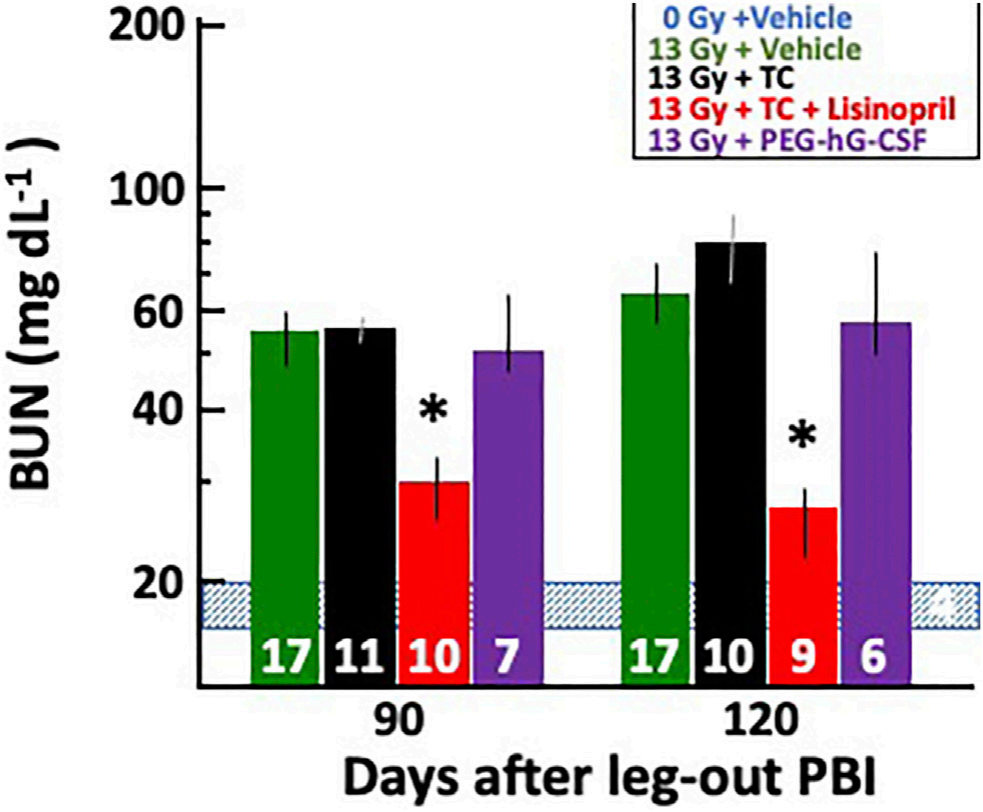
Mitigation of radiation nephropathy after 13 Gy leg-out PBI by lisinopril. The graph shows medians and 20–80% ranges for blood urea nitrogen (BUN in mg dL^−1^) in rats at 90- and 120-days post irradiation. Asterisks (*) represent *p* < 0.05 as compared to irradiation only, irradiation with triple combination (TC) and irradiation with PEG-hG-CSF groups at the corresponding times show increased BUN levels. The normal BUN for non-irradiated rats ranges between 18 and 21 mg dl^−1^ and is represented by the shaded, horizontal blue bar. Numbers in the bars represent N in each group. The decrease in N’s between 90- and 120-days post-irradiation were not due to radiation nephropathy; therefore, they were not given a value of 120 mg dl^−1^.

Blood urea nitrogen (BUN) measurements were used to determine renal injury during radiation nephropathy. Rats in all groups were bled via jugular vein at 90- and 120-days post-irradiation and their median BUNs with 20–80% ranges are plotted in Figure 7. BUNs for the non-irradiated controls are represented by a shaded, horizontal blue bar. All irradiated rats had an increase in BUN values at 90-days which continued to increase by 120-days. The 13 Gy TC + lisinopril group had significantly lower BUNs as compared to the other irradiated rats (*p* < 0.05).

##### Histological Chages at 120-Days to Determine Renal Injury

**FIGURE 8:**
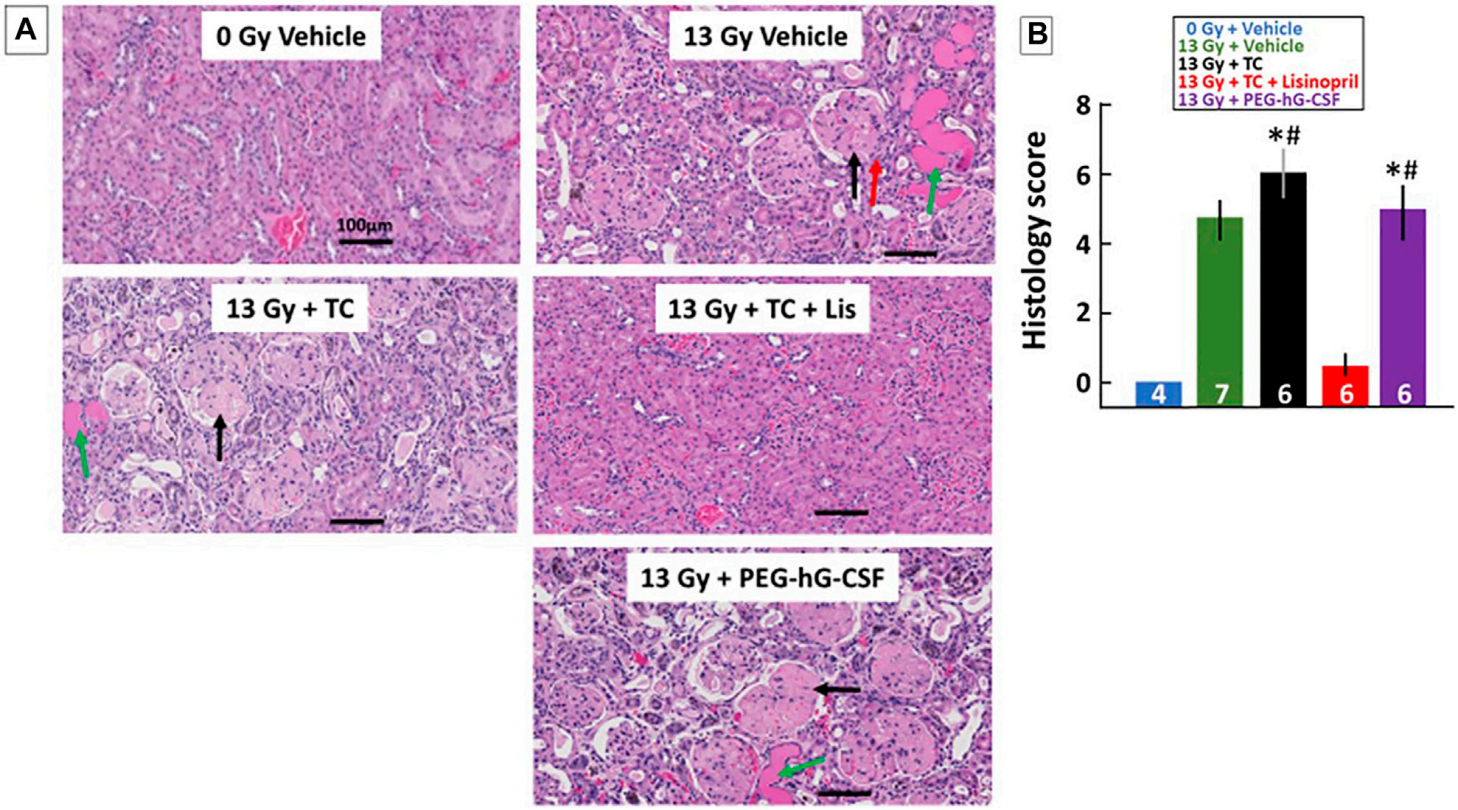
Kidney injury mitigated by lisinopril after 13 Gy leg-out PBI. Representative histological sections of kidneys for each treatment group **(A)** were harvested at the termination of the study (120-days) after 13 Gy leg-out PBI, fixed and stained with H&E. Irradiated rats given the vehicle or PEG-hG-CSF showed increased protein casts (green arrow), glomerular sclerosis (black arrow) and glomerular mesangiolysis (red arrow) compared to non-irradiated rats. The irradiated and triple combination (TC) group showed increased protein casts and glomerular sclerosis while the addition of lisinopril mitigated these histological changes. Graphical representations of the H&E-stained kidney sections are shown in **(B)** as a composite histological score. Renal injury was increased with irradiation, the asterisks (*) represents *p* < 0.05 as compared to non-irradiated controls while the pound symbol (#) represents *p* < 0.05 as compared to the irradiated + TC + lisinopril. Numbers in the bars represent N in each group and bars are means with standard deviation.

Renal histology at 120-days was also used to quantify kidney injury during radiation nephropathy. When the study was terminated at 120-days, kidney sections were stained with H&E and blinded for scoring histological changes (see *Methods*). Figure 8A shows examples of representative histology of all treatment groups. Tissue sections were also given a composite score by the presence of protein casts (green arrow), glomerular sclerosis (black arrow) and glomerular mesangiolysis (red arrow). All irradiated groups showed histological evidence of injury as shown in the graph in Figure 8B by increased composite scores. Irradiated rats that received the TC had a higher composite histology score in comparison to the non-irradiated rats and the irradiated rats that received TC + lisinopril. The irradiated rats that received TC + lisinopril showed mitigation of structural damage in the kidney as compared to the other irradiated groups.

### Mitigation of Delayed Effects of Acute Radiation Exposure to 160 Days

#### Survival After 13 Gy Leg-Out PBI With Lisinopril

**FIGURE 9:**
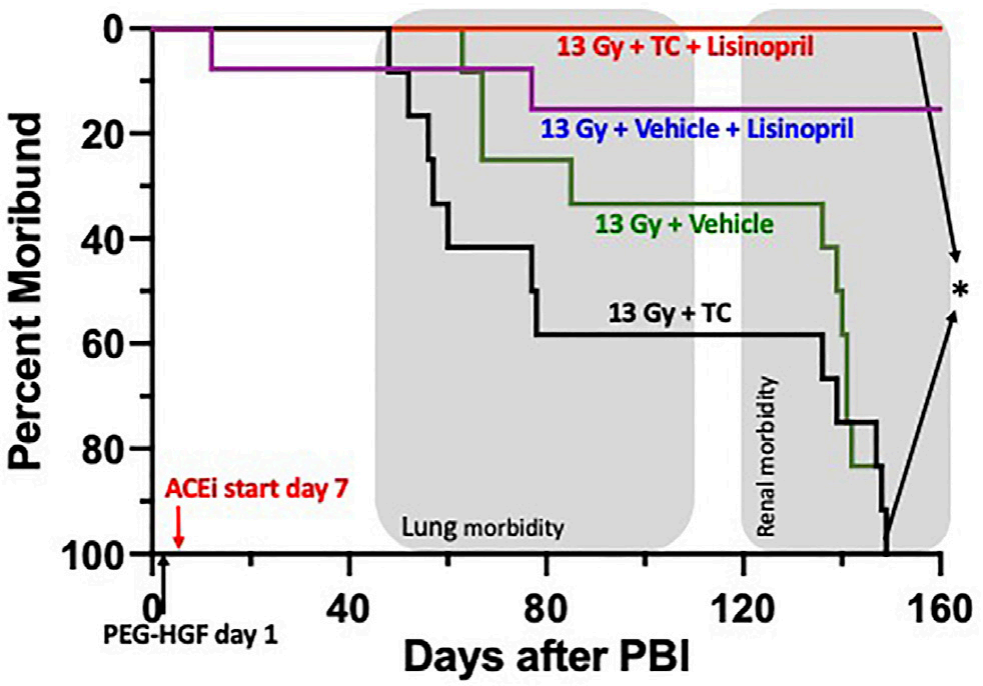
Kaplan-Meier plot representing morbidity from DEARE up to 160 days post 13 Gy partial body irradiation with one hind limb shielded (leg-out PBI). The triple combination (TC, consisting of PEG-hG-CSF, PEG mGM-CSF and PEG hIL-11), or vehicle were given subcutaneously 24 h post PBI and the ACE inhibitor, lisinopril, was started in the drinking water 7-days post-irradiation. All irradiated rats were given supportive care. All irradiated rats that received TC + lisinopril survived to 160 days as compared to 100% morbidity for irradiated rats that received TC alone (*p* < 0.0001). Survival of irradiated rats given vehicle + lisinopril was over 90%, while irradiated rats given only the vehicle were moribund before 160 days. Shaded (gray) areas represent the timing for the lung and renal injuries.

Since renal failure (BUN > 120 mg/dl) occurs beyond 120 days, a separate group of rats were tested for 160-days. Rats were irradiated with 13 Gy leg-out PBI at 11–12 weeks of age and randomly assigned to one of four treatment groups (see *Methods* and Figure 9) to assess survival (primary end point) through 160-days. Figure 9 shows a Kaplan-Meier survival plot for these four treatment groups. By 160-days, all of the irradiated rats given vehicle or TC were moribund. One rat in the vehicle and lisinopril group (Figure 9 **)** was moribund at 11 days, possibly from H-ARS since internal hemorrhaging was observed without obvious GI injury at necropsy. GI lethality usually occurs by 7 days in this model (Fish et al., 2020). The first (lung) sequelae occurred between 60 and 80 days coinciding with pneumonitis (Fish et al., 2016) followed by a second (renal) phase after 140 days (representing radiation nephropathy). Only 1/12 rats in the 13 Gy + vehicle + lisinopril was moribund during pneumonitis while no rats in this group developed severe nephropathy up to 150 days (Figure 9). The experiment was terminated at 160 days at which time survival for all rats given TC + lisinopril was 100% (*p* = 0.0001, 13 Gy + TC + lisinopril vs. 13 Gy + TC).

#### Mitigation of Radiation Nephropathy With Lisinopril

**FIGURE 10:**
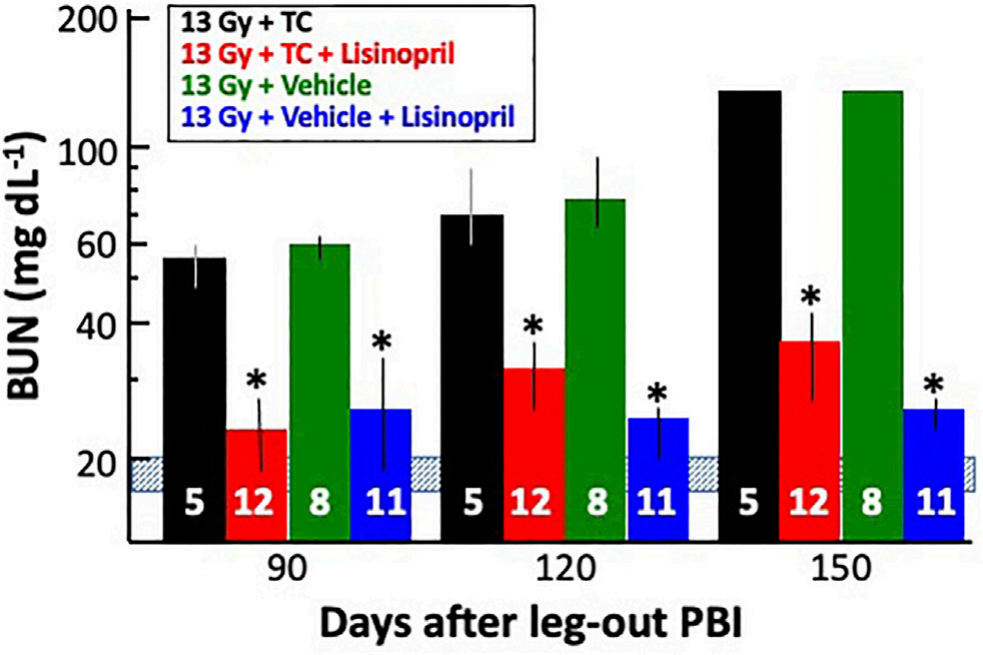
Mitigation of radiation nephropathy after 13 Gy leg-out PBI by lisinopril (∼24 mg m^−2^ d^−1^). The graph shows medians and 20–80% ranges for blood urea nitrogen (BUN in mg dL^−1^) in rats at 90-, 120- and 150-days post-irradiation. Asterisks (*) represent *p* < 0.05 as compared to 13 Gy + TC (triple combination, black bar) at the corresponding times. The normal BUN for non-irradiated rats ranges between 18–21 mg dl^−1^ and is represented by the shaded, horizontal blue bar. All rats with 13 Gy + TC and 13 Gy + vehicle were moribund after 120 days with a BUN ≥ 120 mg dl^−1^ and were given a BUN ≥ 120 mg dl^−1^ at 150 days. Numbers in the bars represent *N* in each group.

Surviving rats in all groups were bled via jugular vein at 90-, 120- and 150-days post-irradiation and their median BUNs with 20–80% ranges are plotted in Figure 10. BUNs for the non-irradiated controls are represented by a shaded, horizontal blue bar. All irradiated rats had an increase in BUN values at 90-days. The BUN of irradiated rats given vehicle or TC continued to increase at 120- and again at 150-days. However, rats given 13 Gy + lisinopril with vehicle or TC had significantly lower BUNs as compared to irradiated rats given TC (*p* < 0.05) indicating non-lethal radiation-induced renal injury.

## Discussion

The goal of these studies was to assess a promising polypharmacy approach of combining a triple combination of growth factors with the ACE inhibitor lisinopril to mitigate ARS and DEARE in two rat models of irradiation (7.5 Gy TBI and 13 Gy leg-out PBI).

### Mitigation of ARS by the Triple Combination in the Presence of Lisinopril

The TC consisting of PEG-hG-CSF, PEG mGM-CSF and PEG hIL-11 mitigated morbidity of the hematopoietic injury of ARS after TBI (Figures 2, 3). The addition of lisinopril to the TC also resulted in increased survival. Survival in TC + lisinopril treated rats was not different from that of TC alone. Thus, even though lisinopril on its own did not mitigate morbidity, it did not alter efficacy of the TC. The advantage of TC to enhance survival during ARS as compared to FDA-approved mitigators such as G-CSF (Neupogen) or GM-CSF (Leukine), is that TC was only injected once vs. multiple injections required for a single non-pegylated growth factor (USFDA, 2015a). Interestingly, recent studies in four mouse strains have found enhanced survival with an abbreviated schedule of G-CSF (Neupogen, 0.17 mg kg^−1^) given for 3 days after irradiation compared to a 16-days schedule (Satyamitra et al., 2017). PEGylated growth factors that possess longer biological half-lives, increased survival to 30-days post-irradiation in irradiated mice after only one treatment (0.1 mg kg^−1^) (Chua et al., 2014). A shortened schedule is an important consideration in the context of a mass casualty event such as a nuclear accident or radiological attack, where single injections would be more convenient than multiple daily injections. Future studies investigating efficacy of a single dose of TC in a second species such as irradiated NHP will be required for approval of TC as a mitigator of H-ARS, via the FDA Animal Rule.

In clinical settings, G-CSF is used by neutropenic patients until neutrophil recovery is achieved (USFDA, 2015a). Similarly, G-CSF, when injected in irradiated mice until day 16, helps to increase neutrophil counts (Plett et al., 2012), likely causing the 35% increase in survival observed in mice. In the current study, complete blood counts taken between 10 and 30-days post-irradiation showed that TC with or without lisinopril enhanced neutrophil counts by 18 but not 10-days as compared to lisinopril alone. Thus, the TC appeared to accelerate recovery of neutrophils after 10-days, suggesting a similarity to the mechanism reported in mice. GM-CSF accelerated neutrophil recovery as a single agent but different injury model in rats (Cox et al., 2020; Singh and Seed, 2020), justifying its use in the TC. Though mGM-CSF has a very fast half-life in rats (terminal half-life was 1.1 h (Cox et al., 2020), the half-life of PEG mGM-CSF is much longer (terminal half-life of 17.2 h (Cox et al., 2020). The dose used in the current study (0.55 mg kg^−1^) is higher than effective doses given to NHP and that used in the clinic (7 μg kg^−1^ day^−1^), though only a single dose was given to rats as compared to multiple doses to NHP (unpublished results) and humans as per dosing recommendations for LEUKINE^®^).

Evidence in mice also suggests hematopoietic growth hormones may protect hematopoietic stem cells by promoting quiescence, thereby maintaining stem cell production (Davis et al., 2008). PEG-GM-CSF and PEG-IL-11 have been shown to cause acceleration of red blood cells and platelet recovery in mice (Plett et al., 2014). PEG-IL-11 increased the induction period of the hematopoietic stem cells compared to IL-11 (Lee et al., 2012; Kumar et al., 2018) and been shown to increase bone marrow cellularity, megakaryocytes, and hematopoietic recovery in mice (Kumar et al., 2018). These mechanisms observed in mice need to be tested in rats, in future studies to confirm the mechanisms of action of TC.

Other studies have shown that the ACE inhibitor captopril increased survival in irradiated mice to day 30 (Davis et al., 2010; Day et al., 2013), though this was not the case for rats (Moulder et al., 1993). The ACE inhibitor captopril has been shown to improve reticulocyte, leukocytes, erythrocytes, and platelet counts in irradiated mice (Davis et al., 2010; Barshishat-Kupper et al., 2011). There were no significant differences in these metrics in this study with lisinopril (results not shown).

Secondary endpoints for GI toxicity were not evaluated because the dose of radiation after 7.5 Gy was well below the threshold to observe external GI injury (seen above 12.5 Gy in WAG/RijCmcr rats, Fish et al., 2020).

### Mitigation of DEARE by Lisinopril in the Presence of TC

Lisinopril has been used to mitigate lung-DEARE in rats after 12.5–13 Gy leg-out PBI (Fish et al., 2016) which was confirmed with secondary endpoints of breathing interval and lung histology. Our current data show that the TC + lisinopril also enhances survival and mitigates the progression of pneumonitis and nephropathy in irradiated rats. Though the mechanism of mitigation of radiation injury is not confirmed, ACE inhibitors are known to benefit cardiovascular function (Goodfriend et al., 1996; Inagami, 1999). This action may play an important role in mitigating radiation-induced injury to well vascularized organs such as the heart, lungs and kidneys. TC alone, which benefits the immune system, but not endovascular injury was not able to mitigate radiation pneumonitis or nephropathy. IL-11 has proven to be an effective mitigator against radiation induced renal injury in mice (Lee et al., 2012). However, we did not find this to be the case with TC which contains IL-11, unless the TC was combined with lisinopril, as indicated by renal histology and BUN levels. It is possible that the benefits of IL-11 were neutralized by the other two growth factors in TC, indicating further experimentation is needed in the future. Lisinopril alone has previously shown to be an effective mitigator against renal injury in the same model as used in this study (Fish et al., 2016). It continued to mitigate nephropathy in rats in the current study in the presence of growth factors that may be used to mitigate ARS; therefore, TC does not interfere with the mitigating effects of lisinopril for DEARE in rats.

Secondary endpoints for GI toxicity were not evaluated to minimize handling of rats in the first 7-days after 13 Gy and because the leg-out PBI dose used (13 Gy) has been described in previous studies to largely spare lethal GI-toxicity (Medhora et al., 2019).

Interestingly, compared to irradiated rats given PEG-hG-CSF (BBT-015) alone, the breathing intervals in irradiated rats given TC + lisinopril were significantly improved in the current study. This is consistent with previous data, which using a different injury model in rats, showed that lung injury is exacerbated by G-CSF (Adachi et al., 2003). Since G-CSF is a component of TC, the results indicate that lisinopril may have efficacy to mitigate pneumonitis in irradiated rats that have been given G-CSF. The TC + lisinopril group also demonstrated lung and renal histology that was not different from non-irradiated rats. TC alone did not improve these metrics as seen by increased vessel wall thickness, alveolar wall thickness and foamy macrophages in the lung, or protein casts and glomerular sclerosis in the kidney (Figures 6, 8). In summary, in a rat model, lisinopril mitigated lung and kidney DEARE in the presence of TC, administered early after radiation, so that the combination was effective for mitigation of both ARS and DEARE.

ACE inhibitors were evaluated in the clinic and reported to improve outcomes for radiation-induced pneumonitis in cancer patients (Jenkins and Welsh, 2011; Kharofa et al., 2012). Other similar clinical studies did not detect clear benefits of these drugs against radiation pneumonitis (Bracci et al., 2016; Small et al., 2018; Sun et al., 2018; Sio et al., 2019). One limitation of these trials was difficulty in accrual which resulted in underpowered analyses. In addition, it is not clear if the consistent high (though approved) doses of ACE inhibitors used in preclinical studies were given to all patients.

### Limitations

There are several limitations of the current study. First, we have not tested each hematopoietic growth factor (PEG-GM-CSF or PEG-IL-11) separately in rats to determine if either of these alone could mitigate ARS or if they were additive. In fact, any one of these factors could even be deleterious by itself, partially neutralizing the beneficial effects of the others. Though mitigation of G-CSF was controversial (Neta, 1988; Neta et al., 1988) each growth factor was tested in mice and enhanced survival as well as hematopoietic cell recovery during ARS (Plett et al., 2014). Second, there was no correction for attrition in the blood count studies. It is uncertain if the results from moribund animals, when included with those from survivors, could further alter blood cell counts and the results presented in Figure 3. Also, as results are different in rats than what has been reported in mice, we do not know how these mitigators will impact humans. Further research must be done in order to determine the efficacy of this polypharmacy approach in other species. Lastly, not understanding the basis for mitigation of each agent is an important limitation. Knowledge of the mechanisms of radiation-induced bone marrow injury in rats will permit better comparison to mice and humans. Mechanistic studies are not in the scope of the current study, the goal of which is to demonstrate for the first time a polypharmacy approach toward mitigating four sequelae arising from irradiation of multiple organs. The individual and combined mechanisms of action for each agent (hydration, antibiotic, each growth factor, and lisinopril) on each sequela must be pursued in future work.

## Conclusion

A triple combination of hematopoietic growth factors (TC) given with the ACE inhibitor lisinopril, successfully mitigated ARS and DEARE in two rat irradiation models used in the current study. The TC + lisinopril group showed decreased morbidity, faster neutrophil recovery and less lung and renal injury, which in some instances was comparable to the non-irradiated control rats. Using PEGylated drugs meant only one administration of hematopoietic growth factors was needed compared to other studies using non-pegylated growth factors requiring multiple injections. Thus, PEGylation is advantageous for a mass casualty accident or attack, as emergency personnel and health care staff would not be needed to deliver repeated dosing. The combination of growth factors and lisinopril was safe and compatible in the rat models and may be an effective medical countermeasure for humans by mitigating acute and delayed injuries in the event of a nuclear disaster or accident.

## Data Availability

The raw data supporting the conclusions of this article will be made available by the authors, without undue reservation.
