# Corrigendum: Polypharmacy to Mitigate Acute and Delayed Radiation Syndromes

**DOI:** 10.3389/fphar.2021.741485

**Published:** 2021-08-25

**Authors:** Tracy Gasperetti, Tessa Miller, Feng Gao, Jayashree Narayanan, Elizabeth R. Jacobs, Aniko Szabo, George N. Cox, Christie M. Orschell, Brian L. Fish, Meetha Medhora

**Affiliations:** ^1^ Department of Radiation Oncology, Medical College of Wisconsin, Milwaukee, WI, United States; ^2^ Department of Medicine, Medical College of Wisconsin, Milwaukee, WI, United States; ^3^ Department of Physiology, Medical College of Wisconsin, Milwaukee, WI, United States; ^4^ Cardiovascular Center, Medical College of Wisconsin, Milwaukee, WI, United States; ^5^ Department of Veterans Affairs, Research Service, Zablocki VAMC, Milwaukee, WI, United States; ^6^ Institute for Health and Equity, Division of Biostatistics, Medical College of Wisconsin, Milwaukee, WI, United States; ^7^ Bolder BioTechnology Inc., Boulder, CO, United States; ^8^ Department of Medicine, Indiana University School of Medicine, Indianapolis, IN, United States

**Keywords:** polypharmacy, acute radiation syndrome, delayed effects of acute radiation exposure, mitigation, hematopoietic growth factor, lisinopril, supportive care, radiation pneumonitis

In the original article, there was a mistake in Figures 2, 4, 9 as published. “The y axis was incorrectly labeled as 0-100, whereas it should be labeled 100-0.” The corrected Figures 2, 4, 9 appear below.

**FIGURE 2:**
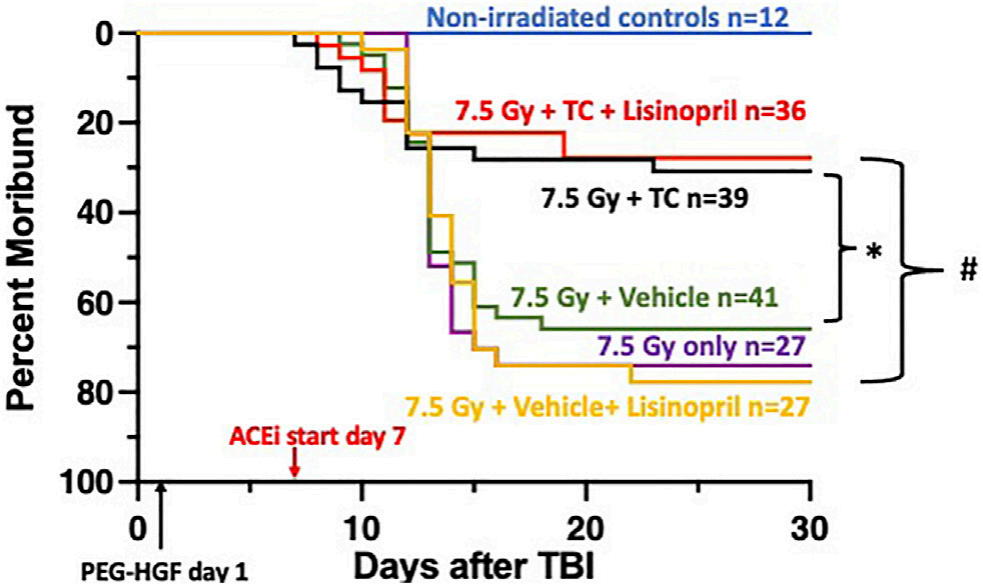
Mitigation of hematopoietic-acute radiation syndrome (H-ARS) by triple combination with and without lisinopril. Kaplan-Meier plots show morbidity through 30-days after 7.5 Gy total body irradiation (TBI). The triple combination (TC, consisting of PEG-hG-CSF, PEG mGM-CSF and PEG hIL-11) or vehicle were given subcutaneously 24-h after TBI (designated by PEG-HGF) and the ACE inhibitor, lisinopril, was started in the drinking water 7 days after irradiation. The number of rats in each group is designated by the “n.” Non-irradiated controls are represented with the blue line. Morbidity was not different in the three irradiated groups given 7.5 Gy only, with vehicle or lisinopril, but survival was enhanced in the group which received the TC (*p* 0.05, denoted by * compared to 7.5 Gy + vehicle group). Survival was increased in the irradiated group receiving TC and lisinopril compared to the irradiated rats receiving the vehicle for TC and lisinopril (*p* < 0.05, denoted by #).

**FIGURE 4:**
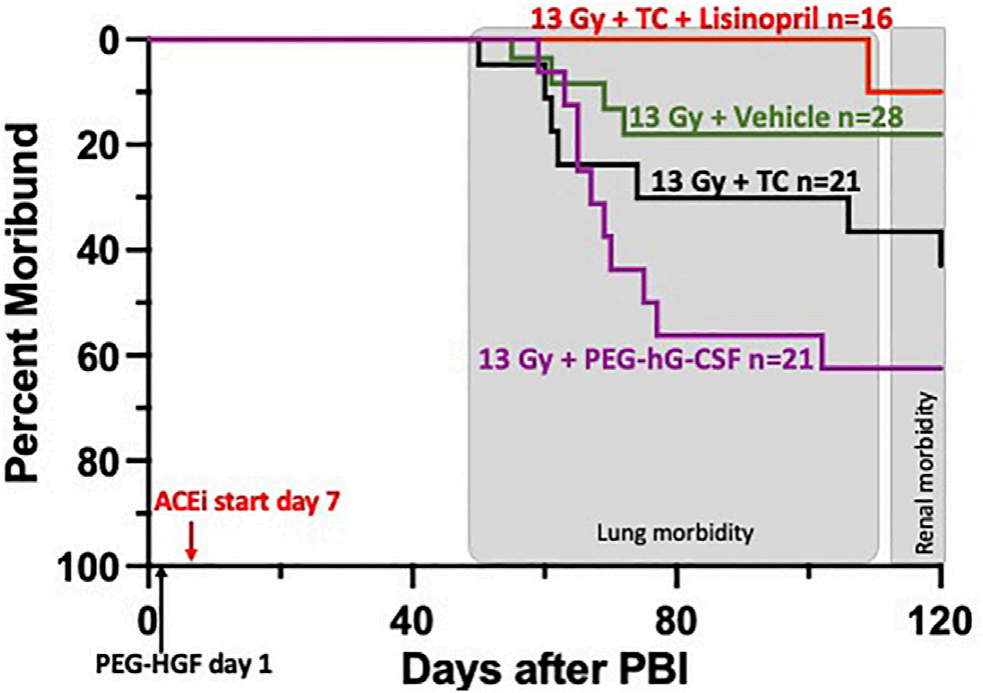
Kaplan-Meier plot representing morbidity from DEARE up to 120 days post 13 Gy partial body irradiation with one hind limb shielded (legout PBI). The triple combination (TC, consisting of PEG-hG-CSF, PEG mGMCSF and PEG hIL-11), vehicle or PEG-hG-CSF (BBT-015) were given subcutaneously 24 h post leg-out PBI (designated by PEG-HGF) and the ACE inhibitor, lisinopril, was started in the drinking water 7 days post irradiation (24 mg m^−2^ d^−1^). All irradiated rats were given supportive care with subcutaneous hydration (40 ml kg^−1^ d^−1^) and enrofloxacin (10 mg kg^−1^ d^−1^) from days 3–7 to 2–14, respectively,. There was trend in lower morbidity in irradiated rats that received TC + lisinopril compared to irradiated rats that received PEG-hG-CSF, but this was not significant (*p* 0.07). Shaded (gray) areas represent the timing for the lung and renal injuries.

**FIGURE 9:**
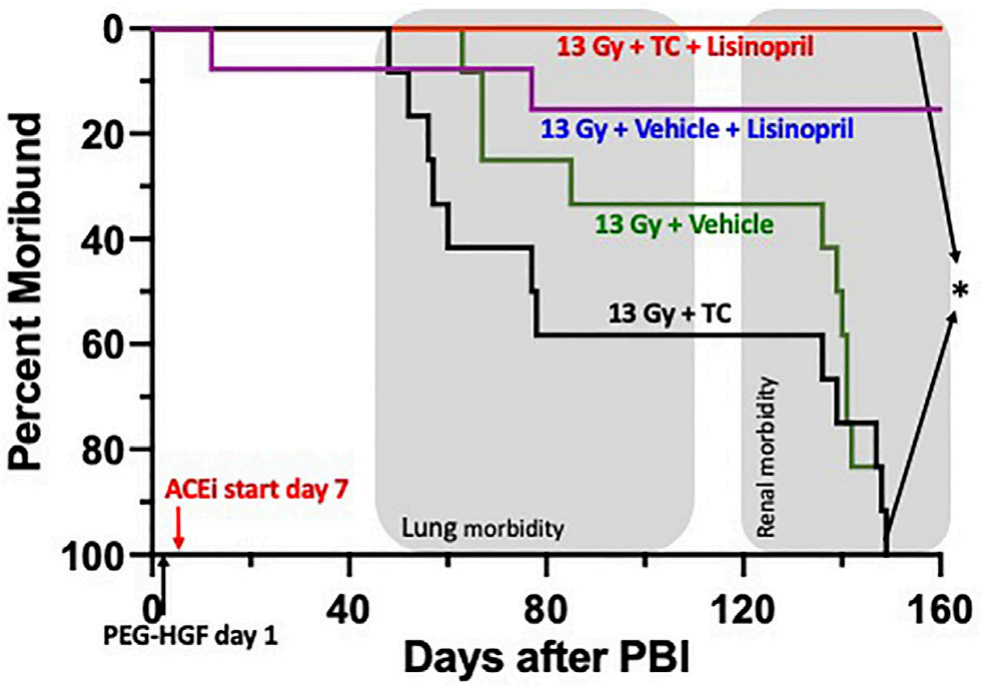
Kaplan-Meier plot representing morbidity from DEARE up to 160 days post 13 Gy partial body irradiation with one hind limb shielded (legout PBI). The triple combination (TC, consisting of PEG-hG-CSF, PEG mGMCSF and PEG hIL-11), or vehicle were given subcutaneously 24 h post PBI and the ACE inhibitor, lisinopril, was started in the drinking water 7-days post-irradiation. All irradiated rats were given supportive care. All irradiated rats that received TC + lisinopril survived to 160 days as compared to 100% morbidity for irradiated rats that received TC alone (*p* < 0.0001). Survival of irradiated rats given vehicle + lisinopril was over 90%, while irradiated rats given only the vehicle were moribund before 160 days. Shaded (gray) areas represent the timing for the lung and renal injuries.

The authors apologize for this error and state that this does not change the scientific conclusions of the article in any way. The original article has been updated.

